# GEROFIT TO HOME (GTH) AS A NEW MODEL OF CARE

**DOI:** 10.1093/geroni/igac059.1630

**Published:** 2022-12-20

**Authors:** Rebekah Harris, Miriam Morey, Elisa Ogawa, Alan Wesley, Jonathan Bean, Katherine Hall

**Affiliations:** VA Boston, Boston, Massachusetts, United States; Durham VA Health Care System, Durham, North Carolina, United States; VA Boston, Boston, Massachusetts, United States; Puget Sound VA, Seattle, Washington, United States; VA Boston, Boston, Massachusetts, United States; Durham VA Health Care System, Durham, North Carolina, United States

## Abstract

Gerofit, a facility-based program transitioned to GTH, a completely virtual structured exercise program, at the onset of the pandemic and previously demonstrated that Veterans already engaged sustained performance with this transition (In-Person vs GTH, n=46; arm curls 19.7 vs 19.0 reps; 30-second chair stand 14.3 vs 15.9 reps). This study investigated whether gains in performance are achieved and sustained in Veterans who participated in GTH only, as it is unknown if virtual programs are as robust as facility-based programs. Measures of performance (3-month mean change, n=45, Arm Curls +3.4 reps; 30-second chair stand +1.5 reps) and self-reported function (SF-36 + 1.8 points) will be assessed from baseline to 3,6, and 12 months. Pooled results from 14 sites have been accumulated and change scores, age and gender-based percentile changes and clinically meaningful thresholds will be presented. This knowledge can have implications to support programs for older adults aging in place.

